# Use of microarray technology to assess the time course of liver stress response after confinement exposure in gilthead sea bream (*Sparus aurata *L.)

**DOI:** 10.1186/1471-2164-11-193

**Published:** 2010-03-22

**Authors:** Josep A Calduch-Giner, Grace Davey, Alfonso Saera-Vila, Benoit Houeix, Anita Talbot, Patrick Prunet, Michael T Cairns, Jaume Pérez-Sánchez

**Affiliations:** 1 Fish Nutrition and Growth Endocrinology Group, Department of Biology, Culture and Pathology of Marine Fish Species, Institute of Aquaculture Torre de la Sal (CSIC), 12595 Ribera de Cabanes, Castellón, Spain; 2 Martin Ryan Institute, National University of Ireland, Galway, Ireland; 3 INRA-SCRIBE, Fish Adaptation and Stress Group, IFR Reproduction, Development and Ecophysiology, Rennes Cedex, France

## Abstract

**Background:**

Selection programs for growth and stress traits in cultured fish are fundamental to the improvement of aquaculture production. The gilthead sea bream (*Sparus aurata*) is the main aquacultured species in the Mediterranean area and there is considerable interest in the genetic improvement of this species. With the aim of increasing the genomic resources in gilthead sea bream and identifying genes and mechanisms underlying the physiology of the stress response, we developed a cDNA microarray for gilthead sea bream that is enriched by suppression substractive hybridization with stress and immunorelevant genes. This microarray is used to analyze the dynamics of gilthead sea bream liver expression profile after confinement exposure.

**Results:**

Groups of confined and control juvenile fish were sampled at 6, 24, 72 and 120 h post exposure. GeneSpring analyses identified 202 annotated genes that appeared differentially expressed at least at one sampling time (P < 0.05). Gene expression results were validated by quantitative PCR of 10 target genes, and K-means clustering of differently expressed genes identified four major temporal gene expression profiles. Set 1 encompassed a rapid metabolic readjustment with enhanced uptake and intracellular transport of fatty acids as metabolic fuels. Set 2 was associated with a wide variety of tissue repair and remodeling processes that were mostly mediated by the stress response of the endoplasmic reticulum (ER). Sets 3 and 4 encompassed the re-establishment of cellular homeostasis with increased intracellular trafficking and scavenging of reactive oxygen species (ROS), accompanied by a bidirectional regulation of the immune system and a general decline of ROS production.

**Conclusions:**

Collectively, these findings show the complex nature of the adaptive stress response with a clear indication that the ER is an important control point for homeostatic adjustments. The study also identifies metabolic pathways which could be analyzed in greater detail to provide new insights regarding the transcriptional regulation of the stress response in fish.

## Background

Selective breeding to ameliorate stress susceptibility has long been established in terrestrial vertebrates of economical relevance [1], but cultured fish are essentially non-domesticated species and selection programs for growth and stress traits are still in their infancy [2,3]. In salmonids, however, a genetic component exists for the stress-mediated response of cortisol [4-6], and selective breeding for low- and high-cortisol responders has been performed in rainbow trout (*Oncorhynchus mykiss*) after handling and exposure to a confinement stressor [7,8]. In carp (*Cyprinus carpio*), a high heritability has also been found for the stress-related increases in plasma cortisol levels after cold shock exposure [9]. Progeny of gilthead sea bream (*Sparus aurata*) from parents selected for low- or high-cortisol response also show divergent responses [10], although in this species low-cortisol responders can be more sensitive to handling and confinement [11]. This finding is indicative that parameters other than plasma cortisol levels must be considered to fully understand the regulation of stress responsiveness and susceptibility. For instance, the cortisol peak is indicative in gilthead sea bream of the intensity and duration of stressor when data found in the literature are compared, but a bimodal rise after acute stress confinement has been reported by some authors [12,13], which would be indicative of some exhaustion of the hypothalamic-pituitary-interrenal axis. By contrast, protein and transcript levels of glucose-regulated protein 75 (GRP75/mortalin), a mitochondrial chaperone of the HSP70 family, are increased by both acute and chronic stress confinement in liver tissue [14]. In addition, transcriptional and promoter analyses of duplicated growth hormone receptors (GHR) indicate that GHR-II rather than GHR-I is a stress sensitive gene in gilthead sea bream [12,15].

With the advent of "omic" technologies, a significant portion, if not all, of the set of transcripts, proteins or metabolites that a study wishes to consider can be determined in one assay. cDNA- and oligo-arrays are in fact powerful tools for the gene expression profiling of many thousands of genes, and various zebrafish (*Danio rerio*), medaka (*Oryzias latipes*) and fugu (*Takifugu rubripes*) microarrays have been developed for toxicogenomics, immunity, development and environmental stress research [16]. Genomic resources in fish species of interest in aquaculture are also continuously growing and species-specific microarrays are now available in salmonids [17-21], flatfish [22,23] and catfish [24,25]. In the case of gilthead sea bream, a highly cultured fish throughout the Mediterranean, a first microarray with 10,176 clones from a cDNA library of embryonic and larval origin has been developed by Sarropoulou et al. [26] to analyze gene expression profile during early development and cortisol treatment. Recently, a second microarray has been constructed and used in developmental studies by Ferraresso et al. [27]. The aim of the current study is to gain more understanding of the physiology of the stress response using a third gilthead sea bream microarray, enriched by subtractive hybridization (SSH) with stress and immunorelevant genes. Time series analyses of data revealed the complexity of the adaptive stress response and underscore the utility of this custom-made array to analyze homeostatic adjustments to stress. This understanding will have practical implications on fish culture and management.

## Results

### Microarray construction

A total of 36,870 gilthead sea bream nucleotide sequences were available for the construction of a cDNA sea bream microarray. Most were sequenced either from tissue EST collections made available by the Consortium of Marine Genomics Europe or from SSH libraries constructed, as described herein, from target tissues after stress and parasite challenges (for details see Construction of SSH libraries in Methods section), and some were obtained from public databases. The number of assembled sequences was 19,069: 12,409 singletons and 6,660 contigs with two or more ESTs in depth [http://www.sigenae.org/aquafirst]. Clone sequences derived from confinement and pathogen SSH libraries [GenBank: FP331359-FP339571, FP339630-FP340170] were represented in 4,851 unique sequences, and most of these (3,161) were novel gilthead sea bream sequences. The entire list of assembled sequences was annotated by BLASTX search on the UniProt database and 6,064 unique sequences showed a significant similarity (E value < 10^-5^) to known genes. Taking into account contigs that were homologous to the same gene, the final number of unique genes on the microarray was 4,876. This platform has been assigned the Gene Expression Omnibus (GEO) accession number GPL8467. It was possible to assign gene ontology (GO) annotations to 6,193 features and the most prevalent GO categories at level 3 (biological processes and molecular functions) are summarized in Table 1.

**Table 1:** Top biological and molecular GO terms (level 3) on the gilthead sea bream cDNA microarray.

GO description (biological process)	Features
Primary metabolic process	1855
Cellular metabolic process	1843
Macromolecule metabolic process	1523
Regulation of biological process	806
Cellular component organization and biogenesis	736
Transport	693
Regulation of cellular process	678
Cell communication	549
Biosynthetic process	484
Multicellular organismal development	481
Anatomical structure development	470
Regulation of metabolic process	374
Cellular developmental process	359
Response to stress	293
Catabolic process	280
Cellular localization	265
Establishment of cellular localization	263
Cell development	261
Anatomical structure morphogenesis	250
Macromolecule localization	230
Establishment of protein localization	209
Generation of precursor metabolites and energy	200
Regulation of biological quality	182
Cell cycle	178
Death	166
Cell proliferation	165
Nitrogen compound metabolic process	159
Response to external stimulus	157
Response to chemical stimulus	146
Cell cycle process	137

**GO description (molecular function)**	

Protein binding	1452
Hydrolase activity	688
Ion binding	666
Nucleic acid binding	562
Nucleotide binding	511
Transferase activity	473
Oxidoreductase activity	342
Substrate-specific transporter activity	247
Signal transducer activity	208
Transmembrane transporter activity	195
Cofactor binding	117
Enzyme inhibitor activity	109
Ligase activity	98
Transcription factor activity	95
Structural constituent of ribosome	93
Lipid binding	84
Enzyme activator activity	74
Lyase activity	64
Isomerase activity	57
Translation factor activity, nucleic acid binding	54
Transcription activator activity	52
Transcription cofactor activity	52
GTPase regulator activity	52
Vitamin binding	51
Tetrapyrrole binding	50

### Expression analysis

**Figure 1:**
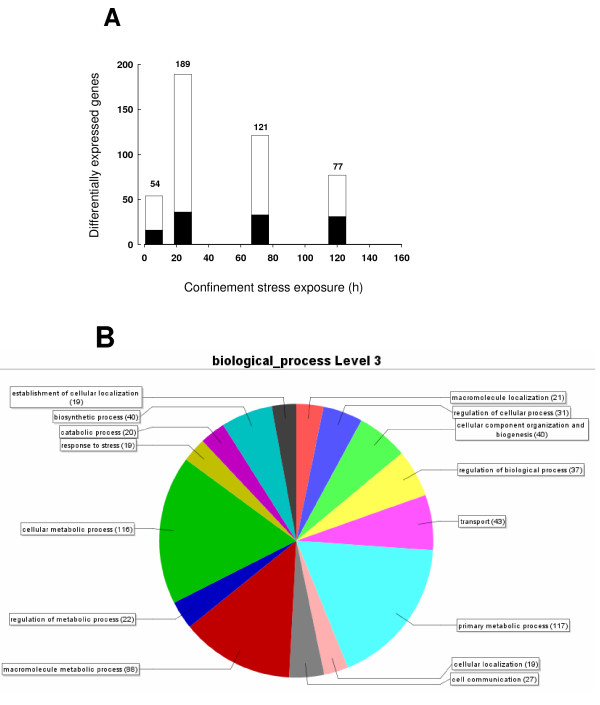
**Differential gene expression in response to confinement exposure (A)**. Time course of differential gene expression. Down-regulated (black bars) and up-regulated (white bars) annotated genes at each time point are represented. The number of differentially expressed genes is indicated at the top of each bar. **(B) **Gene ontology term profile of differentially expressed genes, based on Blast2GO analysis for level 3 of the Biological Process category.

To analyze the time course of the response to stress, fish of 110-130 g body weight were sampled at 6, 24, 72 and 120 h after transfer from 500-l tanks (8-10 Kg/m^3^) to cylinder net baskets of 10-l volume (117-123 Kg/m^3^). Given this body weight,, the rearing density for control fish is near to optimum for gilthead sea bream. The analysis of the hepatic gene expression profile after 10-fold increase in fish density (the confinement stressor) identified 660 unique transcripts as differentially expressed genes (P < 0.05, one-way ANOVA by time and condition). Among these, 202 transcripts were annotated genes, and the time course of changes in up- and down-regulated genes at each sampling point is shown in Figure 1A. After 6 h of confinement, a relative low number of genes (54) were differentially expressed. The maximal number of differentially expressed genes (189 genes) was attained at 24 h, and after that the number of differentially expressed genes decreased progressively to 77 at 120 h. Analysis by Blast2GO revealed the involvement of these differentially expressed genes in a wide variety of processes including among others biosynthetic processes, transport, cell communication, response to stress and regulation of metabolism and catabolism (Figure 1B).

**Figure 2:**
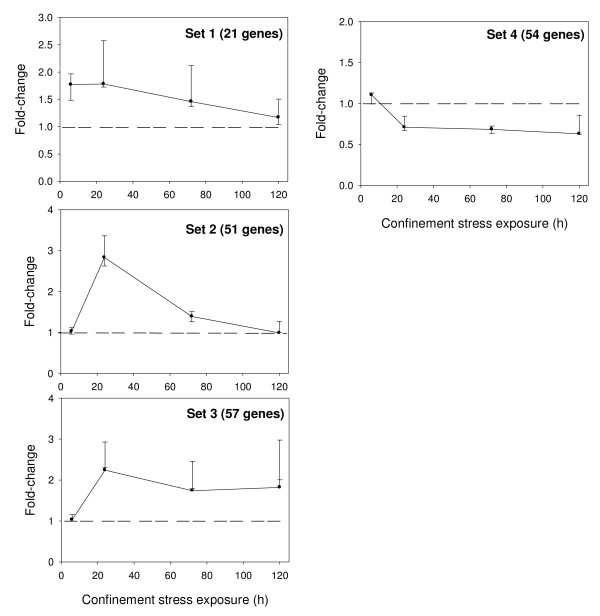
**Fold-change expression profiles of gene clusters**. At each time point, median values (95% confidence interval) of fold-change are represented.

K-means clustering of differentially expressed genes identified four major expression patterns displaying distinct temporal profiles (Figure 2). The first cluster set was composed of 21 genes that showed an early up-regulation at 6 h after confinement. The second cluster set was composed of 51 genes that displayed a delayed and strong up-regulation at 24 h with a fast recovery to control levels by 120 h. The third cluster set was composed of 57 genes with a late but more sustained stress induction that was still evident after 120 h of confinement exposure. The fourth cluster set was composed of 54 genes that showed a persistent down-regulation at 24 h through to 120 h of confinement exposure.

In each K-means cluster, FatiGO analysis for the over-representation of GO-terms suggested the enrichment in some biological processes, although adjusted P-values (Fisher exact test) did not fall in the range of statistical significance (P < 0.05). The entire list of up-regulated genes (sets 1, 2 and 3) is shown in Additional file 1. The most distinctive feature of set 1 is the presence of genes involved in tissue uptake and intracellular transport of fatty acids (lipoprotein lipase, heart-type fatty acid-binding protein, acid cholesteryl ester hydrolase), cysteine metabolism (cystathionine β-synthase, cysteine dioxygenase) and oxidoreductase activity (L-2-hydroxyglutarate dehydrogenase, metalloreductase STEAP2) in addition to a repertoire of genes related to the reset of stress response (protein phosphatase 2C homolog 1), apoptosis (cell death-inducing DFFA-like effector protein C, ecto-ADP-ribosyltransferase 4) and cell proliferation (e.g. BolA-like protein 2, interferon-related developmental regulator 1). Many genes of set 2 are involved in tissue repair and remodeling processes through the enhancement of the protein folding capacity of the endoplasmic reticulum (ER) and ER-associated protein degradation. This set includes among other enzymes and chaperones, derlin-1, ubiquitin-conjugating enzyme E2 N, ubiquitin-protein ligase D3, 94 kDa glucose-regulated protein, and 170 kDa glucose-regulated protein. This cluster also comprises a large set of genes involved in the regulation of transcription and translation (e.g. zinc finger proteins 9, 330 and 479, transcription factor Sp1, threonyl-tRNA synthetase), as well as cholesterol and sterol biosynthesis (arachidonate lipoxygenase 3, Δ14-sterol reductase, diphosphomevalonate decarboxylase, farnesyl diphosphate synthetase and ergosterol biosynthetic protein 28). Set 3 is abundant in inhibitors of protein breakdown (e.g. α-2-macroglobulin, Hsp70-binding protein 1), protein targeting and glycosylation (e.g. signal recognition particle 54 kDa protein, ribophorin I) and vesicle-mediated transport (e.g. clathrin assembly protein complex 1, clathrin heavy chain 1, ras-related proteins Rab-10, 1A and 6A). Also in set 3, a number of genes participate in sterol metabolism, cytoskeleton rearrangements (e.g. coronin-1C, tubulin α-chain), immune response (e.g. complement pathway, cyclosporine A-binding protein, hepcidin) and antioxidant defense through their involvement in iron metabolism (ferroxidase/ceruloplasmin), oxygen transport (hemoglobin subunit β) and glutathione synthesis and recycling (cystathionine γ-lyase, glycine N-methyltransferase, GDH/6PGL endoplasmic bifunctional protein, cytoplasmic isocitrate dehydrogenase).

Down-regulated genes (set 4) are listed in Table 2 and reveal a metabolic readjustment mediated by the depletion of many proteins and enzymes involved in amino acid and nucleotide metabolism (e.g. urate oxidase, aspartate aminotransferase, glycine decarboxylase), glycolysis (fructose-biphosphate aldolase), mitochondrial respiration (NADH-ubiquinone oxidoreductase subunit B14.5b, cytochrome c oxidase subunit IV isoform 2), xenobiotic metabolism (alcohol dehydrogenase 1, glutathione S-transferase 3, cytochrome P450 family, monoamine oxidase, retinol-binding protein II) and immune response through a wide range of processes: acute immune response (α-2-HS-glycoprotein), antigen binding and processing (Ig heavy chain Mem5, histocompatibility complex), lectin pathway (fucolectin-1), histamine metabolism (histamine-N-methyltransferase), and inflammatory and interferon signaling (macrophage migration inhibitory factor, interferon regulatory factor 8).

**Table 2:** K-means clustering of down-regulated genes (Set 4).

Putative Id	Uniprot Accession	Score	Description	Fold change (6, 24, 72, 120 h)
**Set 4 (54 genes): Delayed and persistent down-regulation**				
Peroxisome proliferator-activated receptor-binding protein	Q15648	7E-25	Transcription	-1.08, -1.64, 1.03, -1.28
RNA polymerase B transcription factor 3	P20290	2E-71	Transcription	-1.22, 1.18, 1.01, -2.17
Thioredoxin-binding protein 2	Q9H3 M7	3E-22	Transcriptional repression	-1.17, -1.85, -1.21, -1.08
Probable ATP-dependent RNA helicase DDX48	Q91VC3	1E-140	RNA splicing	-1.16, 1.13, 1.15, -2.17
Cleavage and polyadenylation specificity factor 5	Q7T3C6	1E-125	mRNA processing	-1.21, 1.42, -1.05, -2.56
40S ribosomal protein S25	Q6Q311	5E-30	Translation	-1.14, 1.01, 1.13, -2.63
Translation initiation factor eIF-2B subunit gamma	Q4R6T3	8E-6	Translation	-1.03, -3.85, -2.78, -1.51
*Phospholipase A2*	*P00592*	*1E-31*	*Signal transduction*	*1.09, -1.24, -1.45, -3.03*
*Phospholipase D*	*O17405*	*4E-27*	*Signal transduction*	*1.04, -1.37, -1.21, 1.24*
*Calcium/calmodulin-dependent * *protein kinase type II δ chain*	*Q13557*	*2E-70*	*Signal transduction/regulation * *of cell growth*	*-1.21, -1.77, -1.82, -1.24*
*Proto-oncogene tyrosine-protein * *kinase receptor ret*	*P35546*	*1E-25*	*Signal transduction/development*	*-1.10, -1.11, -1.21, -1.92*
*Translationally-controlled * *tumor protein*	*Q9DGK4*	*2E-57*	*Apoptosis, negative regulator*	*-1.43, 1.16, 1.08, -2.78*
Trypsin	P35034	2E-37	Proteolysis/digestion	-1.12, -1.06, -1.15, -2.94
Elastase-1	Q7SIG3	1E-112	Proteolysis/pancreas	-1.21, -1.49, -1.47, -2.17
α-1-antitrypsin-like protein CM55-MS	O54758	3E-69	Protease inhibitor/extracellular	-1.13, -1.25, -1.52, -1.01
Nucleolar GTP-binding protein 1	Q9BZE4	4E-41	Negative regulation of protein ubiquitination	-1.01, 1.16, -1.05, -3.57
Rotamase 1B	P68106	2E-52	Protein folding/cytoplasm	1.42, -2.63, -2.17, -1.27
Signal sequence receptor subunit α	P45433	1E-101	Protein folding/ER	-1.27, 1.87, 1.01, -3.03
*Adenylosuccinate * *synthetase isozyme 2*	*P46664*	*8E-16*	*Nucleotide biosynthesis*	*-1.03, -1.64, -1.57, -1.64*
*Nicotinamide riboside kinase 2*	*Q9NPI5*	*1E-73*	*Nucleotide biosynthesis*	*-1.30, 1.03, 1.03, -2.78*
*Liver-specific * *uridine phosphorylase*	*Q8CGR7*	*1E-84*	*Nucleotide catabolism*	*1.51, -1.09, -1.72, 2.22*
*Malonate-semialdehyde * *dehydrogenase [acylating]*	*Q07536*	*1E-131*	*Valine & pyrimidine metabolism*	*-1.09, -1.04, -1.45, 1.14*
*Urate oxidase*	*P11645*	*1E-111*	*Purine metabolism*	*-1.08, -1.47, -1.41, -1.60*
*Aspartate aminotransferase*	*P00504*	*0*	*Amino acid catabolism*	*1.16, -1.29, -1.96, 1.18*
*Glycine decarboxylase*	*P15505*	*2E-10*	*Glycine catabolism*	*1.24, -1.36, -1.89, 1.38*
*Neutral and basic amino acid * *transport protein rBAT*	*Q07837*	*6E-85*	*Cystine reabsorption*	*1.62, -1.14, -1.30, -1.22*
*Fructose-biphosphate * *aldolase, muscle type*	*P53445*	*6E-26*	*Glycolysis*	*-1.26, -1.34,-1.61, -2.63*
*Cytochrome c oxidase * *subunit IV isoform 2*	*P80971*	*4E-66*	*Mitochondrial respiration*	*-1.27, -1.29, -1.69, -2.02*
*NADH-ubiquinone oxidoreductase * *subunit B14.5b*	*Q9CQ54*	*7E-19*	*Mitochondrial respiration*	*1.37, -2.22, -1.92, 1.74*
*Hemopexin*	*P02790*	*2E-44*	*Heme transport*	*-1.22, -1.40, -2.08, -1.65*
*Solute carrier organic anion * *transporter family member 1C1*	*Q9EPZ7*	*1E-23*	*Ion transport*	*-1.02, -1.85, -1.39, -1.07*
Alcohol dehydrogenase 1	P26325	3E-92	Oxidoreductase activity/xenobiotic metabolism	-1.07, -2.38, -2.63, -5.55
17-β-hydroxysteroid dehydrogenase 11	Q6AYS8	4E-49	Oxidoreductase activity/xenobiotic metabolism	1.53, -2.63, -1.82, 2.07
Cytochrome P450 1A1	O42457	0	Oxidoreductase activity/xenobiotic metabolism	-1.72, -4.35, -2.00, -1.57
Cytochrome P450 2J1	P52786	3E-69	Oxidoreductase activity/xenobiotic metabolism	1.02, -1.54, -1.06, -1.28
Cytochrome P450 2J2	P51589	7E-63	Oxidoreductase activity/xenobiotic metabolism	1.01, -1.59, 1.02, -1.15
Cytochrome P450 2J6	O54750	2E-87	Oxidoreductase activity/xenobiotic metabolism	1.34, -1.82, -2.00, -2.50
Glutathione S-transferase 3	P26697	2E-63	Oxidoreductase activity/xenobiotic metabolism	-1.15, -1.79, -1.54, -1.89
Monoamine oxidase	P49253	4E-66	Oxidoreductase activity/xenobiotic metabolism	1.28, -1.82, -1.75, -1.59
Retinol-binding protein II, cellular	P50120	1E-51	Oxidoreductase activity/xenobiotic metabolism	-1.23, -3.33, -3.03, -1.10
*α-2-HS-glycoprotein*	*Q9N2D0*	*2E-33*	*Acute immune response*	*-1.13, -1.59, -1.85, -2.17*
*Ig heavy chain Mem5*	*P84751*	*4E-38*	*Antigen binding*	*-1.30, -1.07, 1.03, -2.38*
*B-F histocompatibility * *F10 antigen*	*P15979*	*4E-41*	*Antigen processing*	*-1.15, 1.05, -1.45, -1.52*
*H-2 class II histocompatibility * *antigen, A-K α chain*	*P01910*	*7E-25*	*Antigen processing*	*-1.28, -1.03, -1.30, -1.19*
*Fucolectin-1 (lectin pathway)*	*Q9I931*	*3E-37*	*Complement activation*	*1.04, -1.32, -1.35, -1.89*
*Histamine N-methyltransferase*	*P50135*	*3E-75*	*Histamine metabolism*	*-1.08, -1.69, -1.53, -2.17*
*Macrophage migration inhibitory factor*	*P80177*	*1E-40*	*Inflammatory response*	*-1.37, -1.37, -1.02, -1.48*
*Interferon regulatory factor 8*	*Q90871*	*1E-15*	*Interferon signalling*	*1.01, -1.67, -2.94, 1.28*
Centaurin-α 2	Q9NPF8	3E-28	GTPase activator	-1.23, -1.37, -1.03, -2.22
Collagen α1(I) chain	P02457	4E-68	Collagen component	1.43, -1.82, -1.72, -1.24
Gastrulation-specific G12-like protein	Q9CQ20	4E-18	Microtubule depolymerization, inhibition	-1.01, -1.79, -1.89, -1.08
Plakophilin-3	Q9QY23	8E-26	Cell adhesion	-1.45, -1.67, -1.56, 1.01
Radixin	P26043	1E-24	Actin filament capping	1.32, -3.23, -2.04, -1.83
Transmembrane protein 59	Q9QY73	8E-69	Membrane-bound protein	-1.36, -1.41, -1.25, -2.08

Genes involved in similar pathways or processes are grouped and with the same font (italic/non-italic).

### Real-time PCR validation

**Figure 3:**
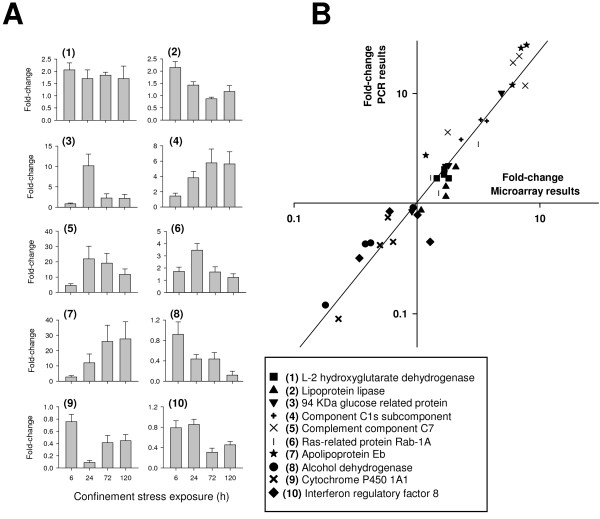
**Real-time PCR validation of microarray results (A)**. Fold-change expression profiles of selected genes determined by real-time PCR. At each time point, bars indicate mean ± SEM (n = 5). **(B) **Correlation plot of fold-change values (control vs stressed fish) for 10 selected genes (at 4 sampling times) analyzed by microarray (X-axis) and PCR methodologies (Y-axis).

The 10 target genes selected for validation of microarray data covered the full range of signal intensity and fold-change results. Their hepatic expression profiles determined by real-time PCR are represented in Figure 3A. In the range of low and intermediate fold-changes, overall data revealed a good correlation between real-time PCR and microarray results (Figure 3B). However, the dynamic range of the microarray is lower than that of the PCR assay, and gene expression changes are under-estimated by the microarray in the upper range of down- and up-regulated changes (0.2 > fold-change > 10), which becomes evident when data on Figure 3B are represented in a linear scale instead of a logarithmic scale.

## Discussion

The SSH technique has been widely used in various animal models to identify tissue-specific or differentially expressed genes between populations of interest [28]. The sensitivity of this methodology is lower than previously envisaged, but in gilthead sea bream this approach has been used successfully for the identification of differentially expressed genes after estradiol treatment [29] and nodavirus infection [30]. In the present study, this technique has also proved successful for the isolation of a relatively high number of novel annotated sequences (more than 20% of the total) which have been used to construct a new gilthead sea bream microarray, contributing to the improvement of genomic resources for a marine fish highly cultured throughout the Mediterranean.

**Figure 4:**
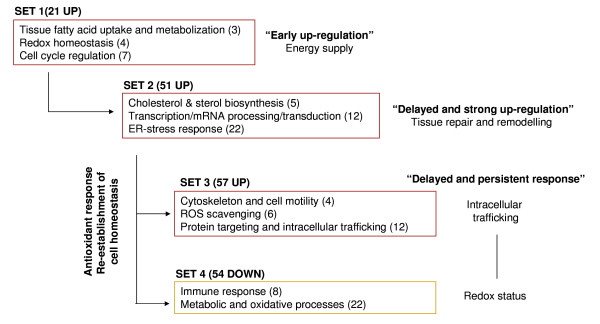
**Enriched-functional groups after confinement exposure in sea bream**. Number of genes involved in each biological process is indicated in parentheses.

In fish and higher vertebrates, the stress response involves changes in plasma glucocorticoids, catecholamines and other classical stress parameters [31,32]. In the case of gilthead sea bream, endocrine and metabolic responses to handling and confinement exposure have been analyzed in some detail [10,33-36], but this is the first attempt at an extensive transcriptional study to monitor the dynamics of the stress response. Given the central role of liver in many homeostatic processes, this organ was targeted for the study and cluster analysis of differentially expressed genes identifying four sets of either up- (sets 1, 2 and 3) or down-regulated (set 4) genes. As summarized in Figure 4, this gene expression profiling shows the complexity of the stress response with early, delayed and persistent adaptive responses which underscore cell-tissue specific adjustments: a) rapid enhancement of energy supply, b) tissue repair and remodeling processes and c) re-establishment of redox balance.

Metabolic rates are increased by stress [37], and the concomitant high energy demand is supported by a rapid mobilization and enhanced tissue uptake of metabolic fuels. Lipids are the most important energy source in carnivorous fish [38,39], and the stress up-regulation of genes involved in lipid transport and fatty acid metabolism points toward the increased availability of energy substrates. At vascular surfaces, lipoprotein lipase (LPL) catalyzes the hydrolysis of circulating triglyceride-rich lipoproteins acting as a limiting enzyme on tissue fatty acid uptake [40]. In gilthead sea bream, the up-regulated expression of LPL is a well documented phenomenon in fish with signs of essential fatty acid deficiencies leading to increase lipid deposition rates in the liver tissue [41,42]. In the present study therefore, the increased expression of hepatic LPL as part of the early stress response was not surprising. Similarly, handling stress also induces the rapid up-regulation of hepatic LPL in rainbow trout [43].

In addition to LPL, other genes involved in lipid metabolism were up-regulated early at 6 h after stress confinement. Acid cholesteryl ester hydrolase, also termed lysosomal acid lipase, hydrolyzes triglycerides and cholesterol esters after lipoprotein endocytosis leading to increases in the cell availability of fatty acids as metabolic fuels. This up-regulation is in agreement with the observations made in mouse and zebrafish models, of enhancement of lysosomal acid lipase by heat shock [44] or hypoxia-associated stress [45]. In the present study, the up-regulation of lysosomal acid lipase coincided with the increased expression of heart-type fatty acid-binding protein. Members of this protein family act as intracellular transporters of fatty acids by trafficking their ligands via interactions with organelle membranes and specific proteins [46]. This transport system ensures a suitable use of, and destination for fatty acids within the cell in each metabolic scenario. Therefore in our model, and perhaps others of increased energy demand, the up-regulation of cytoplasmic fatty acid binding proteins could facilitate the mitochondrial oxidation of fatty acids and may contribute to the protection of the cell against the lipotoxic effects of fatty acids in a changing and oxidative cellular environment. For instance, there are an increasing number of reports that deficiencies or altered functioning of both membrane-associated and cytoplasmic lipid binding proteins are associated with disease states, such as obesity, diabetes and atherosclerosis [47,48].

The transition from normal to stress conditions compromises cell survival and function and this fact could be in concordance with the early induction of apoptotic signals (e.g. cell death-inducing DFFA-like effector protein C, ecto-ADP-ribosyltransferase 4) observed in this study. At the same time, however, the expression of several mitotic and cell proliferation factors (e.g. BolA-like protein 2, interferon-related developmental regulator 1) was enhanced, which could be indicative not only of redundant and overlapping genes but also of complex homeostatic adjustments to stress. Complex trade-off among apoptotic and proliferative factors were also in evidence when genes clustered in sets 2 and 3 were considered. Cell specific adjustments of apoptotic/proliferative pathways seem therefore to be a part of the complex time course of the stress response in gilthead sea bream in order to aid cellular homeostasis. This complex response has been described elsewhere in mammals and other fish species subsequent to toxicological, environmental and stress-disease challenges [49-53].

From our results it can also be concluded that cholesterol and sterol metabolism are transcriptionally mediated by stress confinement in gilthead sea bream. Indeed, several genes annotated with the GO-terms 'cholesterol biosynthetic process' and 'sterol biosynthetic process' belong to set 2 (arachidonate lipoxygenase 3, Δ (14)-sterol reductase, diphosphomevalonate decarboxylase, farnesyl diphosphate synthetase), to set 1 (cytochrome P450 3A10, estradiol 17-β-dehydrogenase 1) and to set 3 (hydroxymethylglutaryl-CoA synthase). Since cortisol is a cholesterol-derived steroid, these transcriptional changes can be viewed as extensive processes aimed to maintain cortisol and steroid production in order to meet the needs of the stress response. As a matter of fact, a recently published study by Saera-Vila et al. [12], with plasma samples coming from the present study, demonstrated a bimodal stress-response of cortisol with a fast and transient peak at 1.5 h followed by a second and more persistent peak at 24 h that would be non-transcriptionally- and transcriptionally-mediated, respectively. The same trend is found when only samples from animals used for microarray analysis are considered (Additional file 2). Similarly, Arends et al. [13] reported a bimodal cortisol response after handling and air exposure, which might highlight some initial exhaustion of the hypothalamic-pituitary-interrenal axis of gilthead sea bream when exposing fish to acute aquaculture stressors.

Also at 24 h after stress confinement, our results suggested the overall activation of transcriptional/translational machinery, possibly as part of the acute stress response to counteract oxidative stress and the associated cellular damage. Thus, chaperones (e.g. 94 kDa glucose-regulated protein, 170 kDa glucose-regulated protein and the co-chaperone Hsp90) are clustered in set 2 and were strongly up-regulated in order to increase the cytoplasmic and ER protein folding capacity. ER chaperones assist the folding of nascent chains and help to achieve an active conformation for mature proteins [54]. In a process referred to as quality control only correctly folded proteins are exported to the Golgi complex, while incompletely folded proteins are retained in the ER to complete the folding process or be targeted for degradation [55,56]. ER-associated protein degradation (ERAD) is, therefore, a process that involves retrotranslocation into the cytosol and proteasome-mediated proteolysis. Ubiquitin, a major player in the proteasomal pathway of protein degradation, is stress activated in fish [57] and ubiquitin mRNA levels are increased after applying a handling stressor in rainbow trout [42]. However, the novel observation from our study is the increased abundance (set 2) of transcripts encoding for proteins involved in ERAD and ER translocation. Of interest later in the time course (set 3) is the dominance of transcripts encoding for extracellular (α-1-microglobulin/bikunin precursor, a-2-macroglobulin) and proteasomal (Hsp70-binding protein 1, RWD domain-containing protein 1) inhibitors of proteolysis in addition to those involved in protein glycosylation, endocytosis and vesicle-mediated transport. Collectively, these findings provide evidence of shifts in protein breakdown and general protein synthesis leading to the complex homeostatic adjustments of protein metabolism to stress. Of particular interest is the up-regulation of clathrin and ras-related proteins which have an accepted role as vesicle-assembly proteins [58-60], although the physiological regulation of these proteins on intracellular protein transport and trafficking has not been reported yet in fish.

The enhanced expression of cytoskeleton and cell motility genes is also part of the late stress response that belongs to set 3. These transcriptional-mediated effects are indicative of a general strategy to generate new cytoskeleton proteins for replacement of the degraded ones, or extensive cytoskeleton reorganization for overall tissue repair and remodeling processes [61]. This adaptive stress response also necessitates the late readjustment of the redox status, which involves a wide range of genes with oxido-reductase activity. Thus, among the genes clustered on set 3, some play a cytoprotective role against oxidative insults promoting the synthesis of cysteine and reducing NADPH equivalents for glutathione synthesis and recycling. In addition, other genes promote the enhanced scavenging of reactive oxygen species (ROS). In this regard, the up-regulated expression of hemoglobin subunit β and ferroxidase, also named ceruloplasmin, was of particular interest. Transcripts levels of α- and β-globin subunits of the hemoglobin (Hb) molecule are increased in gilthead sea bream under extreme farming conditions [62], but the antioxidant effect may also be one of the essential functions of Hb when expressed in the non-erythroid cells of liver tissue. Indeed, the involvement of Hb in the protection of cells against nitrosative stress is a long-time recognized process [63], but Hb can also detoxify highly oxidizing radicals, yielding the ferric state as reported by Nishi et al. [64] in rat mesenglial cells. Likewise, ceruloplasmin is an important regulator of hepatic iron metabolism acting as well as a blood copper-carrier and ROS scavenger [65]. In fish, a cause-effect relationship has not yet been established, but the stress-mediated effects of ceruloplasmin in antioxidant defense are supported by its up-regulated expression not only in this, but also in other models of handling and confinement stress in rainbow trout [20] and of hypoxia-associated stress in cobia [66].

ROS production is inherent to aerobic metabolism and most of the beneficial effects of caloric restrictions are attributed to a reduced oxidative stress [67]. It is not surprising, therefore, that a reduced energy demand is one of the mechanisms operating in the adaptation to chronic stress [68]. For instance, glycolytic enzymes (fructose-biphosphate aldolase) and mitochondrial respiratory electron carriers (cytochrome c oxidase, NADH-ubiquinone) were down-regulated from 24 h through to 120 h (set 4). Liver is also the most important target tissue for detoxifying processes and the depletion of xenobiotic metabolism is also one of the potential ways to reduce ROS production [69]. In the present study, this notion is supported by the down-regulated expression of several enzymes of the cytochrome P450 family, which play a central role in the oxidative metabolism of many endogenous and exogenous substrates [70,71]. Among these, one of the most studied enzymes is CYP P450 1A1, which exhibits reduced expression in the liver of gilthead sea bream with reduced loadings of feed-borne contaminants [72]. Also, reduced expression is found in the liver of common dentex (*Dentex dentex*), a very stress susceptible fish of the Sparidae family, when mRNA transcript levels are compared to those found in gilthead sea bream [73]. Furthermore, our results indicate that retinol-binding protein, a known estrogenic biomarker [74,75], was persistently down-regulated over the course of stress confinement. As far as we know, there is no information in fish about the stress-mediated response of retinol-binding protein. However, the key role of this hepatic protein in fish vitellogenesis is well established [76,77], and its lowered expression could partly explain the impaired fish reproductive performance under confinement and extreme farming [78,79].

Stress is reported to modulate the fish immune functions, although the net effect on the immune system is dependent on the intensity of the stressor. In the short term, acute stress may enhance both cellular and humoral components of fish innate defenses [80-82]. On the other hand, as glucocorticoid hormones such as cortisol have immunosuppressive effects, chronic stress is considered to be detrimental to fish immune function [83,84]. In the present work, stress adaptation after 24 h involves a complicated balance of up- and down-regulation of genes that activate or suppress innate and adaptive immune functions (sets 3 and 4). Similar results have been reported in the liver transcriptome of rainbow trout after 3 h of low-water stress, with a down-regulation of C-type lectins, and up-regulation of major histocompatibility complex components, interferon inducible proteins and complement factors [85]. Bearing in mind the potential that an immune response has as a source of ROS, this profile of expression for sea bream immune genes may reflect not only the maintenance of some specific immune functions, but also the necessity to prevent tissue-oxidative damage associated with a chronic stress condition.

## Conclusions

In the present study we analyzed the time course of the stress response in gilthead sea bream and the results highlight early, delayed and persistent responses which lead to the attainment of a new steady state, and emphasize the complexity of the adaptive stress response. The ER is considered an important control point for the acute and homeostatic adjustments to stress, but later changes in energy demand, oxido-reductase activity and immunological competence are also envisaged. The microarray developed in this study will therefore be suitable for the transcriptional phenotyping of gilthead sea bream welfare and performance, Several studies, for example, are now underway for the multivariate analysis of stress dynamics in relation to nutrition, genotype and stress interactions. Preliminary results are highly promising and practical implications for a more robust and suitable evaluation of new fish feed formulations based on plant ingredients are now possible using these newly established genomic tools.

## Methods

### Experimental setup

Gilthead sea bream juveniles of 10-15 g initial body weight were randomly distributed in eight 500-l tanks in a seawater re-circulatory system equipped with physical and biological filters, and a heat-unit system that maintained water temperature at 18-19°C. Fish grew from July to December at a density of 8-10 kg/m^3 ^with an overall daily growth index [DGI = 100 × (final fish wt^1/3^- initial fish wt^1/3^)/days] of 1.9 ± 0.01. Voluntary feed intake was near to a maintenance ration at the time of the experiment (December), and it was stopped one day before confinement exposure to avoid result disturbances due to differences in feed intake between control (undisturbed fish) and stressed fish. Batches of 10 fish were then transferred from two 500-l tanks to cylinder net baskets of 10-l volume (117-123 Kg/m^3^), suspended each one in 90-l tanks with a high flow of seawater (10 l/min) to avoid water deterioration (Oxygen > 5 ppm; unionized ammonia < 0.02 mg/l). Fish from six additional 500-l tanks were established as undisturbed control groups. No mortality was registered over the course of the experiment. Sampling times for tissue collection were established at 1.5, 3, 6, 24, 72 and 120 h. Eight fish from one control and one confinement tank were netted into a bucket to be anaesthetized with 0.1 g/l 3-aminobenzoic acid ethyl ester (MS-222) (Sigma, Saint Louis, MO, USA). Fish were killed by cervical section and selected tissues (brain, head kidney, gills and liver) were taken, frozen immediately in liquid nitrogen and stored at -80°C. Subsequently, tissue samples were thawed overnight in RNAlater-ICE (Ambion) and were stored at -20°C until RNA isolation. All procedures were carried out according to national (Consejo Superior de Investigaciones Científicas, Institute of Aquaculture Torre de la Sal Review Board) and current EU legislation on the handling of experimental animals.

### RNA isolation

Total RNA was prepared from tissues using a Qiazol and RNeasy Maxi Kit (Qiagen) combination protocol. On-column DNase treatment was incorporated to yield samples predominantly free of contaminating DNA. Total RNA was quantified by spectrophotometric measurements at 260 nm and their quality was analyzed with the Agilent 2100 bioanalyzer (Agilent Technologies). All samples met quality criteria with RNA integrity numbers between 7.5 and 10, indicative of clean and intact RNA.

### Construction of SSH libraries

Tissue samples from the confinement experiment were divided into "early" (1.5 and 3 h sampling time) and "late" (24, 72 h) groups to construct 12 SSH libraries: a) early and late liver libraries, b) early and late head kidney libraries, c) early brain libraries and d) early gill libraries. For each tissue and sampling group, two SSH libraries (forward and reverse) were made from pooled samples of mRNA from control or stressed fish. Four additional SSH libraries were constructed using head kidney and intestine tissue samples from healthy fish and fish infected with the myxosporean parasite *Enteromyxum leei *after 113 days of experimental pathogen exposure [86]. Transcripts of mRNA were purified from total RNA using an Oligotex mRNA Midi Kit (Qiagen). The mRNA populations were quantified by spectrophotometric measurements at 260 nm. They were then analyzed for quality by denaturing agarose gel electrophoresis and probing of Northern blots with the housekeeping gene elongation factor 1-α.

Construction of SSH libraries was performed by means of the BD PCR Select cDNA Subtraction Kit (BD Biosciences Clontech). Two μg of mRNA were used from each sample to generate tester and driver cDNA. As described by the manufacturer, the cDNA was digested with *Rsa *I, tester cDNA was ligated to adaptors and the cDNAs were hybridized and PCR amplified. Aliquots (1.5 μl) of secondary PCR products from subtracted cDNA populations were ligated to the pCR2.1 vector using the TA Cloning Kit (Invitrogen). Aliquots of ligation reactions were transformed into competent Top10 *E. coli *cells. Selected colonies (576-768) from each library were inoculated into 150 μl aliquots of LB kanamycin broth and duplicate glycerol stocks were stored at -80°C. Sequencing of SSH libraries (10,368 clones) was carried out at the Max Planck Institute of Molecular Genetics (Berlin, Germany), using ABI 3730XL (Applied Biosystems) and MegaBACE 4500 (GE Healthcare) capillary sequencer systems. All sequencing reactions were carried out with ABI BigDye Terminator version 3.1.

### Sequence assembly and analysis

cDNA sequences from SSH libraries were combined with other available gilthead sea bream sequences in GenBank and EST collections (29,984 clones) made by the Consortium of Marine Genomics Europe. Sequences were edited to remove vector and adaptor sequences, cleaned and filtered before clustering and annotation by the SIGENAE information system (INRA Toulouse, France). Cleaning involved masking of poor quality bases and low complexity sequences such as polyA tails. Filtering removed contaminating sequences (bacteria, yeast) and only high quality sequences of more than 100 bases in length were retained. Contigs were annotated by comparison to the UniProt database using the BLASTX program http://www.ncbi.nlm.nih.gov/BLAST/, and the entry to which they received the highest similarity was assigned as the gene identity. Gene ontology (GO) identifiers were obtained from the contig nucleotide sequences through the Blast2GO software [87] with a threshold cutoff at 10^-3^.

### PCR amplification and array printing

The cDNA inserts of selected clones were amplified by PCR using adaptor-specific primers. Amplicons were then spotted onto Schott Nexterion slide E epoxy slides at the Max Planck Institute facilities. Each microarray consisted of PCR products from 18,490 cDNA clones printed in duplicate at 5 μm resolution.

### RNA labeling and hybridization

For microarray screening of the time course stress response, liver total RNA from 5 stressed and 5 control animals at each sampling time (6, 24, 72 and 120 h) after confinement exposure were individually analyzed. A reference design approach was taken for the study: all experimental samples were labeled with one of the two dyes (Cy5 or Cy3) and compared to a common reference sample (a pool of all the RNA samples used in the design) labeled with the second dye. Each hybridization experiment included dye swaps to compensate for cyanine dye effects, making a total of 80 microarray slides for the entire analysis. Denatured samples of total RNA (10 μg) were reversed transcribed and indirectly labeled with Cy5 or Cy3 dyes using the SuperScript III Indirect cDNA Labeling System Kit (Invitrogen). Hybridizations were performed in a Genetix hybridization chamber and washes used the Advawash automatic washing station (Advalytix). Microarray slides were scanned in two channels (543 & 633 nm) at 5 μm resolution using a confocal laser scanner (ScanArray Express, Perkin Elmer).

### Microarray data analysis

Two channel TIFF images were imported into the GenePix Pro 6 software program for feature (spot) finding and alignment using a batch alignment process. Features were flagged as present, absent or bad by this software program and pixel intensities for feature and background were quantified. Output GenePix results (GPR) files were imported into GeneSpring GX 7.3. Data was normalised for signal, dye swap and intensity (intensity dependent lowess normalisation) and log 10 ratios of signal (experimental) to control (reference) channel were calculated. Data were filtered on confidence and signal strength. This experiment was placed in the NCBI Gene Expression Omnibus (GEO) as accession number GSE16633. One-way ANOVA by time and condition was performed to select those genes that were differentially expressed (P < 0.05) between control/stress at least at one sampling point over the time course; expression profiles for these genes were clustered over the time course using the K-means algorithm. FatiGO software [88] was used to assess whether specific biological processes or molecular functions were over-represented in each cluster when compared to the others.

### PCR validation

Microarray results were validated by real-time PCR of 10 target genes, selected to reflect the K-means clusters of up- and down-regulated genes. cDNA was synthesized from 2.5 μg of total RNA and 500 ng of polydT primer in a reaction volume of 40 μl, using SuperScript III reverse transcriptase (Invitrogen) following the supplier's protocol. Primers were designed for β-actin (housekeeping gene) and candidate genes (see Table 3) using Vector NTI Advance software (Invitrogen). The size of amplicons ranged between 100 and 150 base pairs and PCR reactions were set up as follows: 10 μl of QuantiTect SYBR Green PCR Master Mix (Qiagen), 1 μl of 10 μM sense and antisense gene-specific primers, 5 μl of cDNA template at a dilution of 1:12.5 and RNase-free water to a final volume of 20 μl. The MX3000P Real-time PCR system (Stratagene) was used for performing the amplification. The program used for PCR was 95°C for 15 min, 40 cycles of 95°C for 15 s, annealing at 51-60°C for 30 s and extension at 72°C for 30 s. Dissociation curves were examined at the end of the PCR reaction to check for unspecific amplification and primer-dimers.

**Table 3:** Primer sequences for quantitative PCR validation.

Gene name	Primer sequence
L-2 hydroxyglutarate dehydrogenase	F AAG GTC TTC ACA ATG ACA ATG GCG
	R TCC CTC GCC ATC GCT GAA AT

Lipoprotein lipase	F TTT ACG CTC TGT GAG GTC TCC GG
	R GGG ACG TTG CCA AGT TTG TGA C

94 KDa glucose related protein	F AGA ACG TGG CAA AGG AGG GTG T
	R TGT CCT TCA GGG CCT TGT CCT T

Complement C1s subcomponent	F CCC ACC CAG TGA TGA CTC CTG A
	R GGC TTC CAG AAC CGA TCT GAC TG

Complement component C7	F TTG ATT CCT GAC AGA CGG TCC CC
	R CGG CTC AAC TCC ACC ACG TTT ACT T

Ras-related protein Rab-1A	F GCT GAA ATC AAG AAG AGG ATG GGC
	R GAG TAA GAG GGC GGG GTG TCA A

Apolipoprotein Eb	F ACT GAA CCA CTA AAA GTG CCC TTC T
	R TAG CCG CAG GAC GTG CAT TTA

Alcohol dehydrogenase	F GTG CTG CAG TTT ATG GGA ACC AGT A
	R TAT TGA CTG CTG CTC CGT ATC CTG T

Cytochrome P450 1A1	F GCA TCA ACG ACC GCT TCA ACG C
	R CCT ACA ACC TTC TCA TCC GAC ATC TGG

Interferon regulatory factor 8	F TGG AGG CAG TGA ACA TGC GG
	R GGG CAT GTT GTC CTT GTA GCA GG

β-Actin	F CTG GAG AAG AGC TAT GAG CTG CCC
	R GGT GGT CTC ATG GAT TCC GCA G

To assess PCR efficiency, standard curves were created by serial dilution of cDNA preparations. Expression of the different samples was normalized to β-actin, and the efficiency of PCR reactions for target and reference genes varied between 88% and 95%. Relative changes in the expression of candidate genes were calculated by the ΔΔC_t _method [89].

## Authors' contributions

JPS and PP conceived and designed the project. JACG, ASV, and JPS designed and performed the confinement experiment and sampled the experimental animals. GD and BH purified RNA from tissues and produced SSH libraries. JACG, GD, and BH performed microarray experiments. GD and MTC carried out all GenePix and GeneSpring analyses. JACG and JPS performed gene ontology analyses. ASV and AT validated array data by qRT-PCR. JACG and JPS wrote the manuscript. All listed authors edited the manuscript. All authors read and approved the final manuscript.

## Supplementary Material

Additional file 1 **K-means clustering of up-regulated genes (Sets 1, 2, 3)**. Genes involved in similar pathways or processes are grouped and with the same font (italic/non-italic). Click here for file

Additional file 2 **Plasma cortisol levels of control (open circles) and stressed (filled circles) fish**. Data are the mean ± SEM (n = 5). Different letters indicate statistically significant changes over the course of the experiment in stressed fish (ANOVA, P < 0.05). Statistically significant differences between stressed and control fish were analyzed at each sampling time by means of Student t-test (*P < 0.05, **P < 0.01, ***P < 0.001). Click here for file
