# Supplementary material for: Use of microarray technology to assess the time course of liver stress response after confinement exposure in gilthead sea bream (Sparus aurata L.)
Source: BMC Genomics. 2010 Mar 22;11:193. doi: 10.1186/1471-2164-11-193 (PMC2860363; doi:10.1186/1471-2164-11-193)
Supplement: Additional file 1 — K-means clustering of up-regulated genes (Sets 1, 2, 3). Genes involved in similar pathways or processes are grouped and with the same font (italic/non-italic). [file 1471-2164-11-193-S1.PDF]

Putative Id	Uniprot Accession	Score	Description	Fold change (6, 24, 72, 120 h)
**Set 1 (21 genes): Early up-regulation**				
Histone H3-K4 methyltransferase	Q8WTS6	1E-161	Transcription	1.40, 2.06, 1.37, 1.55
LINE-1 reverse transcriptase homolog	P08548	3E-15	Reverse transcriptase	1.23, 1.78, 1.25, 1.13
Cell death-inducing DFFA-like effector protein C	*Q96AQ7*	*9E-54*	*Apoptosis/cytoplasm*	*3.03, 5.39, 3.90, 1.10*
Ecto-ADP-ribosyltransferase 4	*Q95NE0*	*3E-16*	*Apoptosis/membrane*	*1.83, 3.26, 1.96, 2.75*
Protein phosphatase 2C homolog 1	*P35182*	*1E-11*	*Reset stress response*	*2.48, 1.67, 2.15, 1.17*
Meiotic recombination protein REC8-like 1	*O95072*	*3E-11*	*Meiosis*	*1.85, 1.42, 1.16, 1.18*
BolA-like protein 2	*Q8BGS2*	*2E-28*	*Cell proliferation*	*2.05, 3.16, 1.81, -1.12*
Interferon-related developmental regulator 1	*Q5S1U6*	*1E-21*	*Cell proliferation*	*1.55, 1.67, 1.06, 1.71*
Pescadillo	*P79741*	*1E-114*	*Cell proliferation*	*1.77, 1.98, 1.02, 1.01*
Collagen α-2 (VIII) chain	P25067	2E-10	Cell adhesion/structural constituent	1.38, 1.57, 1.24, 1.07
CD209 antigen-like protein D	Q91ZW8	5E-21	Probable pathogen-recognition/endocytosis	1.05, 2.17, 1.81, 1.50
Gamma-aminobutyric acid receptor-associated protein-like 1	Q8R3R8	3E-31	Microtubule/intracellular/ authophagic vacuole	1.25, 2.09, 1.31, -1.33
Cystathionine ß-synthase	*P35520*	*4E-97*	*Cysteine synthesis*	*2.42, 2.54, 1.18, 1.26*
Cysteine dioxygenase type 1	*Q6NWZ9*	*1E-100*	*Cysteine oxidation*	*2.17, 2.62, 1.42, 1.80*
L-2-hydroxyglutarate dehydrogenase	*Q9H9P8*	*3E-35*	*Oxidoreductase activity*	*1.91, 1.56, 1.69, 1.62*
Metalloreductase STEAP2	*Q8BWB6*	*2E-34*	*Oxidoreductase activity*	*1.84, 2.69, 3.99, 2.32*
Acid cholesteryl ester hydrolase	P38571	6E-78	Lipase activity/receptor mediated endocytosis/lysosome	1.50, 1.78, 1.83, 1.56
Lipoprotein lipase	P11602	1E-172	Lipase activity/plasma membrane	2.07, 1.72, 1.08, 1.72
Heart-type fatty acid-binding protein	O13008	3E-56	Intracellular fatty acid transport	1.24, 1.99, 2.36, 1.38
Cytochrome P450 3A10	*Q64148*	*5E-8*	*Hydroxylation of steroids*	*1.47, 1.65, 1.67, 1.44*
Estradiol 17-ß-dehydrogenase 1	*P14061*	*2E-37*	*Estrogen biosynthesis*	*1.48, 2.10, 1.46, 1.25*
**Set 2 (51 genes): Delayed and strong up-regulation**				
Transcription factor Sp1	*P08047*	*2E-22*	*Transcription*	*-1.07, 3.07, 1.19, 1.68*
Transcription initiation factor IIE subunit ß	*P29540*	*3E-84*	*Transcription*	*1.11, 1.40, 1.03, 1.02*
Zinc finger protein 330	*Q9Y3S2*	*1E-105*	*Transcription*	*1.07, 1.59, 1.11, -1.33*
Zinc finger protein 479	*Q96JC4*	*5E-31*	*Transcription*	*1.14, 1.80, 1.05, 1.20*
Zinc finger protein 9	*Q3T0Q6*	*1E-65*	*Transcription/sterol mediated repression*	*1.14, 1.74, 1.13, -1.32*
ATP-dependent RNA helicase DDX39	*Q8VDW0*	*1E-108*	*mRNA processing*	*-1.14, 2.84, 1.38, 1.11*
Putative pre-mRNA-splicing factor ATP-dependent RNA helicase DHX15	*O35286*	*3E-60*	*mRNA processing*	*1.04, 3.75, 2.89, 1.61*
Splicing factor, arginine/serine-rich 3	*P84104*	*3E-50*	*mRNA processing*	*1.16, 2.47, 1.22, -1.24*
Eukaryotic peptide chain release factor subunit 1	*P62498*	*6E-22*	*Translation*	*1.87, 3.80, 1.64, 1.21*
Protein pelota homolog	*Q7ZWC4*	*1E-100*	*Translation*	*1.17, 3.08, 1.67, 1.15*
Threonyl-tRNA synthetase	*Q3ZBV8*	*4E-49*	*Translation*	*-1.05, 2.51, 1.29, 1.41*
Developmentally-regulated GTP-binding protein 2	P55039	2E-52	Signal transduction	-1.20, 2.41, 1.28, -1.39

Protein separation anxiety	Q9NHD5	1E-68	Cell proliferation/DNA replication	1.00, 2.94, 1.29, 1.12
Sestrin-2	P58004	6E-31	Cell cycle arrest/nucleus	1.13, 1.73, 1.92, -1.58
Nucleolar protein family A member 2-like protein	Q6PBV6	4E-49	Ribosome biogenesis	1.15, 1.98, 1.37, -1.33
cAMP-regulated phosphoprotein 19	*Q712U5*	*2E-46*	*Post-translational modification/protein catabolism*	*-1.01, 2.41, 1.40, 1.33*
Trypsin-1	*P35031*	*1E-122*	*Proteolysis*	*1.45, 3.82, 1.88, 1.11*
Dipeptidyl aminopeptidase-like protein 6	*Q9Z218*	*3E-97*	*Proteolysis/integral to membrane*	*-1.11, 3.59, 1.11, 2.67*
Hyaluronan-binding protein 1	*Q3T0B6*	*2E-36*	*Proteolysis/secreted/tissue repair and remodeling*	*1.66, 3.25, 1.80, -1.08*
Protein transport protein Sec61 subunit ß	Q5RB31	5E-22	Protein translocation in the ER	-1.21, 3.06, 1.10, -1.30
Transmembrane emp24 domain-containing protein 9	Q3T133	7E-52	Protein translocation in the ER	-1.02, 4.08, 1.22, 1.37
Signal recognition particle 14 kDa protein	P37108	7E-39	Protein translocation and targeting of secretory proteins to ER	1.30, 2.83, 1.49, -1.37
Derlin-1	Q5R9W3	3E-17	ER-associated protein degradation	-1.23, 2.29, 1.10, -1.04
ER-associated Hsp40 co-chaperone	Q9UBS4	2E-93	ER-associated protein degradation	-1.04, 8.70, 1.80, 1.79
RING finger protein 175	Q8N4F7	5E-26	ER-associated protein degradation	-1.37, 1.97, 1.44, -1.83
Ubiquitin-conjugating enzyme E2 N	Q9EQX9	1E-77	ER-associated protein degradation	1.12, 1.91, 1.15, 1.03
Ubiquitin-protein ligase	P61078	4E-80	ER-associated protein degradation	1.57, 3.07, 1.37, 1.50
UBX domain-containing protein 2	Q92575	4E-69	ER-associated protein degradation	1.19, 2.78, 2.06, 1.48
170 kDa glucose-regulated protein	Q60432	5E-98	Unfolded protein response/ER	-1.04, 3.36, 1.45, 1.86
94 kDa glucose-regulated protein	Q29092	1E-132	Unfolded protein response/ER	-1.10, 4.87, 1.81, 1.71
Glucosidase 2 subunit beta	P14314	2E-79	Unfolded protein response/ER	-1.05, 2.30, 1.40, 1.24
Protein disulfide-isomerase 4/rearrangement of -S-S- bonds	P13667	1E-156	Unfolded protein response/ER	-1.25, 7.08, 1.60, 2.79
UDP-glucose ceramide glucosyltransferase-like 1	Q6P5E4	2E-7	Unfolded protein response/ER	1.12, 3.84, 1.39, 2.05
Hsp90 co-chaperone	Q15185	3E-40	Unfolded protein/Cytoplasm	1.01, 2.95, 1.14, -1.34
Fukutin	Q8R507	4E-29	Protein modification/Golgi	1.09, 2.59, 1.25, 1.65
Glutaminyl-peptide cyclotransferase	Q16769	3E-22	Protein modification/acyltranferase	-1.04, 1.82, 1.28, 1.38
Protein Chibby (PKD2 interactor)	Q9Y3M2	1E-33	Intracellular protein transport	1.04, 2.07, 1.68, 1.0
SEC13-like protein 1	Q9D1M0	1E-135	Intracellular protein transport	1.15, 4.08, 1.81, 1.43
Endoplasmic reticulum-Golgi intermediate compartment protein 1	Q4V8Y6	3E-65	Vesicle-mediated transport	-1.33, 3.18, 1.22, 1.64
KDEL endoplasmic reticulum protein retention receptor 2	Q6PEH1	2E-87	Vesicle-mediated transport	-1.11, 3.10, 1.66, -1.25
Vesicular integral-membrane protein VIP36	Q12907	6E-12	Vesicle-mediated transport	-1.23, 2.51, 1.30, 1.04
Arachidonate lipoxygenase 3	*Q9BYJ1*	*2E-47*	*Cholesterol & sterol biosyntheses*	*-1.21, 2.36, 2.00, -1.05*
Δ (14)-sterol reductase	*Q8WMV1*	*1E-115*	*Cholesterol & sterol biosyntheses*	*-1.34, 2.73, 2.19, 1.14*
Diphosphomevalonate decarboxylase	*Q62967*	*5E-66*	*Cholesterol & sterol biosyntheses*	*-1.41, 4.62, 1.82, -1.68*
Farnesyl diphosphate synthetase	*P08836*	*1E-106*	*Cholesterol & sterol biosyntheses*	*-1.57, 5.00, 1.84, -1.65*
Probable ergosterol biosynthetic protein 28	*Q5R589*	*1E-57*	*Sterol biosynthesis*	*-1.89, 3.39, 1.74, -1.37*
Long-chain-fatty-acid--CoA ligase 1	*P33121*	*1E-81*	*Fatty acid metabolism*	*1.14, 2.01, 1.48, 1.18*
Acidic mammalian chitinase	Q91XA9	4E-58	Carbohydrate hydrolysis	-1.49, 2.25, 1.42, 1.21
Arsenical pump-driving ATPase	O54984	2E-99	Anion transport/membrane	-1.13, 1.83, 1.16, 1.15
ATP-binding cassette sub-family F member 2	Q9UG63	1E-98	Nucleotide transporter/mitochondria	1.44, 3.27, 1.35, 1.01
Gluconate kinase 1	P39208	1E-8	Carbohydrate metabolism	1.37, 3.12, 1.83, 1.15

Set 3 (57 genes): Delayed and persistent up-regulation

Cyclic AMP response element-binding protein 2	*P18848*	*4E-23*	*Transcription activation*	*1.21, 2.15, 1.66, 1.45*
Probable ATP-dependent RNA helicase DDX5	*Q5R4I9*	*1E-111*	*mRNA processing/nucleus*	*1.20, 2.24, 1.25, 1.83*
Vigilin	*P81021*	*4E-85*	*mRNA stabilization/cytoplasm*	*1.14, 1.82, 1.20, 1.96*
Elongation factor 1-alpha	*Q9YIC0*	*2E-82*	*Translation*	*1.21, 1.56, 1.45, 1.93*
Nucleolar phosphoprotein p130	*P41777*	*8E-35*	*Translation*	*1.13, 2.58, 1.37, 1.44*
Phospholipase C-γ-2	P16885	8E-17	Signal transduction	-1.19, 2.22, 1.86, 1.93
Phospholipase A-2-activating protein	Q9Y263	1E-95	Signal transduction	1.04, 2.22, 1.86, 1.15
Mitogen-activated protein kinase 6	Q5F3W3	9E-8	Apoptosis inhibition/cytoplasm	1.46, 2.68, 1.31, 2.11
Nucleolysin TIAR	Q01085	3E-32	Apoptosis induction/nucleolysis	-1.38, 2.69, 3.20, 1.59
Cathepsin B	*P07858*	*9E-13*	*Proteolysis/lysosome*	*-1.10, 1.75, 1.36, 1.81*
Hyaluronan-binding protein 2	*Q6L711*	*2E-36*	*Proteolysis/secreted*	*-1.66, 1.81, 3.02, 4.71*
α-1-microglobulin/bikunin precursor	*P00978*	*3E-16*	*Protease inhibitor/secreted*	*-1.25, 1.94, 1.08, -1.64*
α-2-macroglobulin	*P01023*	*1E-130*	*Protease inhibitor/secreted*	*1.06, 1.22, 1.12, 2.19*
Hsp70-binding protein 1	*Q6IMX7*	*4E-29*	*Proteasome inhibitor*	*1.1, 1.94, 1.91, 1.73*
RWD domain-containing protein 1	*Q99ND9*	*8E-77*	*Proteasome inhibitor*	*1.21, 1.69, 1.18, 2.11*
Signal recognition particle 54 kDa protein	Q6AYB5	1E-107	Targeting of secretory and membrane proteins to ER	-1.15, 1.84, 1.37, 1.28
Signal recognition particle receptor subunit beta	P47758	3E-17	Targeting of secretory and membrane proteins to ER	-1.0, 4.20, 1.41, 2.08
Glycoprotein-processing glucosidase I	Q80UM7	1E-91	Protein glycosylation/ER	1.21, 2.67, 1.53, 1.66
Ribophorin I	P04843	3E-65	Protein glycosylation/ER	-1.04, 3.33, 2.24, 2.54
Clathrin assembly protein assembly protein complex 1	Q9BXS5	6E-75	Vesicle-mediated transport	-1.1, 2.31, 1.56, 1.29
Clathrin heavy chain 1	P49951	7E-48	Vesicle-mediated transport	1.03, 2.17, 1.45, 1.42
Coatomer subunit ß	P23514	1E-126	Vesicle-mediated transport	1.43, 2.79, 1.77, 1.78
Protein transport protein Sec23B	Q9D662	1E-74	Vesicle-mediated transport	1.85, 2.52, 1.17, 1.52
Ras-related protein Rab-10	P61027	1E-61	Vesicle-mediated transport	1.10, 1.80, 1,28, 1.18
Ras-related protein Rab-1A	Q05974	1E-106	Vesicle-mediated transport	1.34, 3.77, 1.93, 1.76
Ras-related protein Rab-6A	Q5RAV6	1E-102	Vesicle-mediated transport	1.71, 2.71, 1.65, 1.63
TBC1 domain family member 10A	P58802	5E-66	Vesicle-mediated transport	-1.29, 2.14, 1.74, 2.23
Apolipoprotein Eb	*O42364*	*5E-93*	*Lipid transport*	*1.18, 5.97, 7.03, 7.79*
ATP-binding cassette sub-family A member 1	*P41233*	*3E-80*	*Cholesterol homeostasis/ assembly of HDL*	*1.12, 1.68, 1.49, 1.93*
Hydroxymethylglutaryl-CoA synthase	*P23228*	*4E-93*	*Cholesterol and sterol biosyntheses*	*-1.08, 2.93, 1.99, 1.81*
Cystathionine γ-lyase	P32929	1E-58	Cysteine synthesis/antioxidant defence	-1.01, 2.89, 2.35, 1.91
Glycine N-methyltransferase	P13255	3E-65	Cysteine synthesis/antioxidant defence	-1.07, 1.70, 1.83, 1.49
GDH/6PGL endoplasmic bifunctional protein	Q8CFX1	9E-42	Pentose-phosphate shunt/ NADPH/antioxidant defence	1.06, 2.07, 1.58, 1.73
Ferroxidase/ceruloplasmin	P13635	2E-69	Iron storage/antioxidant defence	-1.05, 1.93, 1.48, 2.34
Hemoglobin subunit ß	Q6Y239	2E-34	Oxygen transport/antioxidant defence	-1.22, 1.98, 2.58, 3.95
Isocitrate dehydrogenase [NADP] cytoplasmic	Q9Z2K8	1E-174	NADPH /antioxidant defence	1.25, 2.51, 2.20, 1.60
Complement C1s subcomponent (classical pathway)	*Q69DK8*	*3E-7*	*Complement activation*	*-1.04, 2.86, 3.31, 3.73*

Complement component C7 (classical & alternative pathways)	*Q9TUQ3*	*2E-54*	*Complement activation*	*1.79, 7.53, 7.06, 9.13*
Complement component C9 (classical & alternative pathways)	*P79755*	*1E-43*	*Complement activation*	*1.15, 2.45, 2.06, 3.13*
Complement factor B (alternative pathway)	*P04186*	*2E-25*	*Complement activation*	*-1.18, 2.24, 1.66, 1.31*
Complement factor H (alternative pathway)	*P08603*	*2E-25*	*Complement inactivation*	*1.18, 1.91, 1.14, 1.65*
Complement factor I (classical & alternative pathways)	*Q9WUW3*	*4E-24*	*Complement inactivation*	*1.72, 3.16, 2.30, 4.17*
C-type lectin domain family 4 member D	*Q8WXI8*	*7E-19*	*Immune response/endocytic receptor*	*-1.32, 1.35, 1.53, 1.15*
Cyclosporin A-binding protein	*P54985*	*9E-66*	*Immune suppressive activity*	*-1.06, 2.63, 1.84, 1.35*
Hepcidin	*P82951*	*7E-34*	*Anemia of inflammation*	*-1.07, 4.14, 5.39, 8.57*
Leukocyte cell-derived chemotaxin 2	*O62644*	*1E-9*	*Chemotaxis/cell differentiation and repair*	*-1.18, 2.82, 3.15, 3.36*
Coronin-1C	Q9ULV4	1E-97	Actin cytoskeleton /cytokenesis/motility	1.02, 2.17, 1.23, 1.52
Extracellular matrix protein 2	O94769	2E-16	Cell-matrix adhesion	1.45, 2.96, 2.20, 1.52
Gap junction Cx32.2 protein	P51915	8E-38	Cell communication	1.39, 4.50, 3.86, 3.94
Tubulin α chain	P30436	0	Microtubule-based movement	1.00, 1.45, 1.13, 1.45
6-Pyruvoyl tetrahydrobiopterin synthase	*Q90W95*	*1E-66*	*Zinc ion binding*	*1.42, 5.29, 3.22, 2.73*
Ancient ubiquitous protein 1	*Q9Y679*	*1E-21*	*Acyltransferase activity*	*-1.22, 1.31, 1.58, 1.28*
Cation transport regulator-like protein 1	*Q5SPB6*	*1E-43*	*Protein binding*	*-1.60, 4.70, 3.40, 7.13*
Proteoglycan-4 precursor (Megakaryocyte-stimulating factor)	*Q9JM99*	*9E-60*	*Catalytic activity/extracellular*	*1.06, 1.61, 1.15, 1.52*
Rhamnose-binding lectin	*Q9PVW8*	*2E-37*	*Sugar binding/secreted*	*-1.24, 2.11, 2.26, 2.18*
Type II antifreeze protein	*P05140*	*9E-29*	*Response to freezing/sugar binding*	*-1.40, 2.94, 1.80, 2.37*
Xylulose kinase	*Q3MIF4*	*1E-31*	*D-xylose metabolism*	*-1.61, 2.12, 3.51, 5.40*
