# Supplementary figures and images for: Use of microarray technology to assess the time course of liver stress response after confinement exposure in gilthead sea bream (Sparus aurata L.)
Source: BMC Genomics. 2010 Mar 22;11:193. doi: 10.1186/1471-2164-11-193 (PMC2860363; doi:10.1186/1471-2164-11-193)

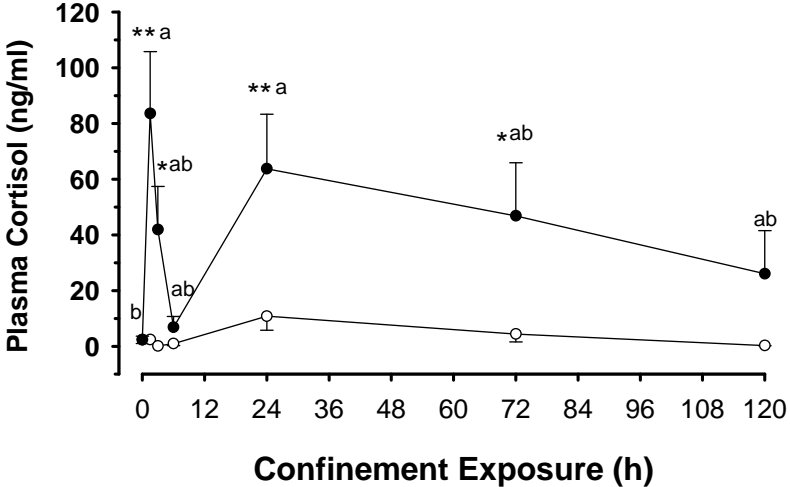

Supplement: Additional file 2 — Plasma cortisol levels of control (open circles) and stressed (filled circles) fish. Data are the mean ± SEM (n = 5). Different letters indicate statistically significant changes over the course of the experiment in stressed fish (ANOVA, P < 0.05). Statistically significant differences between stressed and control fish were analyzed at each sampling time by means of Student t-test (*P < 0.05, **P < 0.01, ***P < 0.001). [file 1471-2164-11-193-S2.PDF]
